# Editorial: Technology innovations for violence prevention, mental wellness and resilience among youth

**DOI:** 10.3389/fdgth.2024.1365726

**Published:** 2024-02-19

**Authors:** Christine Wekerle, Amanda K. Gilmore, Shannon Self-Brown

**Affiliations:** ^1^ Department of Pediatrics, Offord Centre for Child Studies, McMaster University, Hamilton, ON, Canada; ^2^ Department of Psychiatry & Behavioural Neuroscience, Offord Centre for Child Studies, McMaster University, Hamilton, ON, Canada; ^3^ Optentia Research Unit, South-West University, Vanderbijlpark, South Africa; ^4^ Mark Chaffin Center for Healthy Development, School of Public Health, Georgia State University, Atlanta, GA, United States

**Keywords:** technology, prevention, violence prevention, mHealth, youth, mental health, resilience, intervention

**Editorial on the Research Topic** Technology innovations for violence prevention, mental wellness and resilience among youth

## Introduction

Digital mental health refers to the use of digital, mobile, and connected technologies that can be integrated into standard clinical services, utilized in support of long wait-lists, and function as stand-alone interventions in both the prevention and treatment domains (1). There are many challenges within the digital health arena, yet the potential for increased access to mental health and resilience services adds merit to the well-planned research approach to investigate issues from the acceptability to uptake to impact of the digital intervention. This Research Topic arose from the prior work and interest of the three guest editors, Drs. Self-Brown, Gilmore, and Wekerle. Dr. Shannon Self-Brown developed a “Special Issue” in the journal *Child Maltreatment* in 2017 entitled “Technology 2.0: A focus on the newest technological advances in child maltreatment research” (2). Particularly relevant were the innovations to support positive parenting and to prevent maltreatment that utilized e-health avenues. Self-Brown et al. showed that incorporating a digitally-supported content delivery approach into an evidence-based positive parenting program delivery was highly feasible, and significantly reduced program providers' session preparation and time in family sessions, while increasing levels of program fidelity (3). Providing a lower-cost and more time-efficient strategies to support the workforce and participants in positive parenting programs can yield broad reach and increase the impact of prevention efforts on child resilience trajectories.

The extensive need for evidence-based intervention that can be self-initiated was informed by a large data collection study from Ontario child welfare-system involved youth; the “Maltreatment and Adolescent Pathways (MAP)” project [e.g., (4–9)] that indicated their victimizations, especially child sexual abuse and adolescent dating violence, were potentially unreported to the youths' child welfare workers. The work of guest editor, Dr. Gilmore, points to the value of leading novel research on the utility of digital interventions to prevent sexual assault and related behaviors such as substance use among youth and emerging adults. Dr. Gilmore has found that digital prevention programs developed for teens and emerging adults which target both substance use, and sexual assault prevention have demonstrated usability (10) and preliminary efficacy at changing perceptions and behavior (11, 12). Further, with colleagues, Dr. Gilmore has evaluated the efficacy of post-sexual assault interventions with technology-based delivery including video interventions during a sexual assault medical forensic exam in the emergency department to girls and women (13) and treatment for military sexual trauma-related posttraumatic stress disorder (14), suggesting that technology-based secondary prevention is also an effective methodology to reach survivors. Combined, self-paced technology-based interventions are a viable method in preventing sexual assault and reducing the burden of sexual assault in a manner that is both usable and effective.

The work of Dr. Wekerle has explored additional technology innovation approaches that can be self-initiated or incorporated with clinical programming. Her work focused on the design, examination, and dissemination of the JoyPop™ app—an app designed with a focus on promoting resilience among youth who have experienced adverse childhood experiences (ACEs)—includes a trauma-informed care perspective that prioritizes choice and safety (15). Recent studies, including two published in this Research Topic, support the clinical significance of the app (16, 17). Furthermore, recent projects demonstrate the promise of the app for very hard-to-reach populations, such as Indigenous youth (15), and the eagerness of clinical professionals who deliver evidence-based programs, such as TF-CBT and SafeCare, to ACES-impacted youth to have apps like JoyPop™ to supplement program delivery (18, 19). Collectively, the related, yet distinct, emerging work from these guest editors prompted a call to the field requesting emerging research focused on Technology Innovations emerging in the areas of Violence Prevention, Mental Wellness and Resilience among Youth. Papers accepted for this Research Topic clustered around developmental periods and critical themes to move the field forward in our approaches to meeting the needs of ACES impacted youth.

## Opportunities for digital technology to impact youth violence prevention, mental wellness, and resilience

Indeed, relationships are the learning “ground” for both psychopathology and well-being across the lifespan. Self-righting is a natural, adaptive developmental process to seek out, engage with, and otherwise galvanize internal processes of self-determination. Within positive, “corrective” relationships, the autonomous person tends towards self-correction and healthy pathways. Within the child maltreatment field, two recognized developmental “windows” of opportunity to self-right relational patterns and behaviors are adolescence and parenthood, given the new context of adaptation challenge (20). Adolescence, as a developmental period of rapid maturation, is often associated with various physical, environmental, and psychosocial stressors. However, there are opportunities for relational resilience, with the developmental push for both autonomy and affiliation (Knapp et al.). Parenthood is another opportunity to revise dysfunctional interactional patterns, and enhance relationship repair skills (21). Among the six articles in this Research Topic, three are related to adolescent and young adult resilience (Knapp et al.; Malik et al.; Maurer et al.), and three are related to the parenting context (Baggett et al.; Prokos et al.; Tiwari et al.), which will be further elucidated in the next two sections.

## Adolescent and young adult digital health resilience

Although most research related to adolescent and young adult mental health focuses on mental health symptoms and disorders, mental health resilience is an important field as it can improve the lives of adolescents and young adults through a different but related perspective. Three papers in this Research Topic (Knapp et al.; Malik et al.; Maurer et al.) focus on adolescent and young adult mental health resilience using digital health interventions and strategies. Maurer et al. and Malik et al. focus on the usability and preliminary efficacy of a digital health resilience intervention, JoyPop™, while Knapp et al. discuss findings from qualitative interviews suggesting that the public library may be a viable implementation infrastructure for digital mental health services for adolescents.

Maurer et al. and Malik et al. focused on the JoyPop™ smartphone app, which focuses on improving resilience among youth and young adults. Malik et al. examined the usability of JoyPop™ among six adolescents aged 12–17 and seven service providers. Maurer et al. assessed the preliminary efficacy of JoyPop™ with social work participants who used the app two times daily over a 28-day period; 2-week and 4-week follow-ups noting changes in affect regulation, stress responsivity, and social support were conducted. Approximately one-third of the sample were exposed to adverse childhood experiences. These findings revealed significant reductions in affect dysregulation and perceived stress, especially among those who were exposed to four or more adverse childhood experiences. Taken together, these studies suggest preliminary usability for adolescents and service providers, as well as preliminary efficacy among young adults.

Knapp et al. examined the role that a public library can play in adolescent resilience. Using interviews with 17 library workers, they found that library workers are dedicated to providing digital mental health services to adolescents, providing a safe space for adolescents, and addressing the mental health needs of historically underrepresented racial and ethnic adolescents. Importantly, the public library is a resource that supports patrons through free services and the infrastructure may be an important place to leverage to implement digital mental health services, especially for adolescents from marginalized backgrounds.

To summarize, this Research Topic highlights three important phases of research within digital health resilience for adolescents and young adults including assessing usability, testing preliminary efficacy, and understanding factors associated with implementation. The intervention and implementation infrastructure assessed here can have wide uses. If the JoyPop™ app is found to be efficacious in a large randomized controlled trial, it has the potential for widespread and cost-effective implementation due to the intervention delivery modality of an app. Further, the public library infrastructure has a number of existing structures in place to implement digital health resilience programs to adolescent youth, especially those from historically underrepresented racial and ethnic backgrounds. Continued work in these areas is warranted as further research can have a significant, positive impact on adolescent and young adult mental health resilience.

## Digital health and child maltreatment programming

Addressing child maltreatment as a public health concern requires innovative solutions, and as seen in the 3 relevant papers in this Research Topic, digital innovations can provide tailored support for maternal well-being, reducing the risk of child abuse, addressing disparities in services, fostering positive parenting behaviours (Baggett et al.; Tiwari et al.), and may be feasible for injury prevention (Prokos et al.). Baggett et al. leveraged data from a recent trial with 184 Black mothers of infants experiencing high levels of economic distress and low social support, who participated in digital interventions targeting depression and parenting to explore program engagement. High levels of parent engagement were reported, far exceeding engagement rates in home visiting programs, which are currently considered the gold standard for child abuse prevention. There was also a lower risk of child maltreatment for mothers who completed at least 10 digital sessions. This demonstrates that virtual interventions for new, at-risk mothers are highly feasible, as they have the potential to overcome access and service barriers associated with individuals particularly vulnerable period for maternal depression and child maltreatment risk.

A second paper by Prokos et al. shared lessons learned from an ongoing trial of a 5-week child injury prevention program for families living in poverty that was transitioned from in-person to virtual delivery in the context of the pandemic. Modifications that were made to the study included use of: participant cell phones to conduct data collection and intervention sessions; virtual meeting software to conduct sessions with participants; and an online platform to collect questionnaire data. In terms of feasibility, the investigators were able to collect all of the data that was originally proposed; however, recruitment and retention was more challenging. The authors reported numerous lessons learned, including logistical barriers such as providing technology devices and problem-solving connectivity issues so that families could continue intervention and assessment participation. Another learned lesson reported by the authors that has relevance for the broader virtual delivery of parenting programs is that when completing parenting intervention sessions virtually, it is important to coordinate with the participant to discuss session engagement while there are multiple children in the home, as parent supervision often understandably led to service disruption. Authors concluded with recommendations for ensuring success in virtual intervention studies with families at-risk, noting that multimodal instructions can be helpful, and that provision of devices and Wi-Fi are still sorely needed when working with low-income individuals.

Lastly, Tiwari et al. conducted a scoping review inclusive of 25 peer-reviewed articles and two peer reviewed abstracts to explore technology utilization in evidence-based parenting and child programs serving families impacted by child maltreatment following the onset of the pandemic, which created unique opportunity for program implementation via technology due to social distancing restrictions. Two main uses of technology emerged: (1) remote programmatic delivery (i.e., delivering all or part of the program virtually using technology) and (2) programmatic enhancement (i.e., augmenting program content with technology). Importantly, review findings amongst parents and children across the 11 studies measuring participant satisfaction on the use of both telehealth and explored digital enhancements were generally encouraging, noting favorable reception or satisfaction rates among users, which may be a proxy indicator of perceived quality or engagement. Similar to Baggett et al., these findings across studies were encouraging in exploring innovative technologies and the mechanisms that promote behavioral change that will serve to reduce child maltreatment risk and deleterious outcomes for those impacted by maltreatment.

Collectively, these studies demonstrate both feasibility and generation of positive outcomes for vulnerable families participating in digitally-delivered, child-maltreatment focused interventions. Although these advancements have shown positive results in improving behavioural outcomes for parents and children, the digital divide remains an issue. In this context, it is crucial to address these equity issues to ensure that all vulnerable families can access this technology. Upon addressing issues pertaining to the digital divide, the research above suggests that digital programming can greatly reduce other access inequities that are a hallmark of other clinical or home-delivered evidence-based programs. Continued work is warranted to ensure digital programs can lead to a public health impact on child maltreatment.

Ultimately, this Research Topic highlights that technology can be an innovative resource for supporting self-determination, better health, and resilience amongst the youth population. However, socioeconomic factors associated with technological accessibility play a vital role in the resource's success. This was specifically seen during the COVID-19 pandemic, as there was a focus in adapting technological use in clinical settings for low-income families to best support their needs (Prokos et al.). This involved providing greater access to various technologies, such as phones and internet access to complete online programs. In connecting technology to youth resilience, there must be an incorporation of various determinants of health, as this informs future resource pathways, intervention implementation, and youth wellbeing.

## Connections to youth

Digital mental health services, including initiatives in public libraries, aim to expand access to mental health care, reducing barriers such as stigma and health disparities in low-income communities (Knapp et al.). Libraries, as community hubs, align with the rights principles, and governmental responsibilities, by providing equal opportunities. The core values of library workers are pivotal in advancing children's best interests, and are in the position to curate teen digital mental health resources. In turn, involving the community in digital mental health services caters to marginalized youth's unique needs. One area of support may be the connection to the librarian as a resource person for understanding the effectiveness and match-to-need of a digital resource, web-based or mobile phone application. Mobile mental health applications proliferate with few developed in consultation with and targeted to youth. Findings suggest that youth can benefit from digital tools as adjuncts or stand-alone support, and may be especially useful in regions that are under-served by mental health and children's healthcare systems; these formats may function as “just-in-time” interventions (1).

Two articles present user response to a new youth resilience mobile application designed to address emotion regulation skills. Resilience, a key component of children's well-being, is strengthened by focusing on increasing social, individual, and environmental factors that reduce the likelihood of an individual's exposure to adversity (Malik et al.). Digital technology, like JoyPop™, have a significant potential to enhance the resilience of young individuals, especially those who have been exposed to ACEs. JoyPop™ is specifically designed for adolescents, with a focus on promoting resilience through strengthening protective factors, particularly emphasizing self-monitoring, self-awareness, and emotional regulation (Malik et al.; Maurer et al.). This aspect of the app empowers adolescents to actively manage their mental health, instilling a sense of autonomy and control that is fundamental to their overall wellbeing. Further, the app's central focus on emotion regulation and transdiagnostic treatment takes a similar approach of the CRC, which emphasizes the comprehensive well-being of children (Malik et al., Maurer et al.). In addition, the app's commitment to assisting adolescents in overcoming adversity is linked to the recognition of a child's inherent right to positive mental and social development.

## Future directions in digital health research focusing on violence prevention, mental wellness, and resilience of youth

In closing, recognizing children's unique vulnerabilities and the long-term impact of trauma points to continued efforts to address youth risk and resilience at the family- and individual-level. Digital innovations can play a pivotal role in addressing these concerns. Specifically, digital mental health services are a method to provide expanded access to mental health care and health disparities in low-income communities. Digital innovations can create tailored and engaging support to promote youth well-being, reducing the risk of detrimental trajectories of those impacted by trauma, and preventing revictimization and/or violence perpetration. One important aspect of digital interventions is that they can be tailored; tailoring based on gender identity, race, ethnicity, and sexual orientation when indicated could be leveraged to help reduce existing health disparities. Future approaches can also support parental behaviour allowing for both individual and relational approaches for promoting youth health by disseminating evidence-based relational digital content.

Moving forward, it is imperative to continue investigating and implementing technological innovations that positively impact the lives of vulnerable youth. In doing so, there should be a greater focus on overcoming technological disparities, increasing access to technology and promoting equitable access, in the context of well-planned research programs. A model of mobile health practice outlines five broad research areas of interest: (1) the evidence base from providers, the healthcare system, the client (child, parent, family); (2) the clinical integration process to existing service structures, involving training; (3) privacy and security issues; (4) ethical considerations (e.g., within the mobile app's drop-down menu for 24/7 helplines); and (5) cultural considerations (e.g., special populations, youth subculture, language-match capacities etc.). Digital health innovation research may be helpful to circle back to theory and mechanisms of action to further direct adaptations and updates towards greater utility and impact. With the ubiquity of devices, especially within child development today where social relationships and education are primarily integrated with digital delivery in some form, it is an exciting period to continue developing, testing, and disseminating digitally-delivered health focused initiatives.
